# A new species of *Paratanais* Dana, 1852 (Crustacea, Peracarida, Tanaidacea, Paratanaidae) from Puerto Rico, northwestern Atlantic

**DOI:** 10.3897/zookeys.397.6137

**Published:** 2014-04-03

**Authors:** Andrés G. Morales-Núñez, Richard W. Heard

**Affiliations:** 1 Department of Natural Sciences, University of Maryland Eastern Shore, Princess Anne, MD 21853,USA; 2 Department of Coastal Sciences, University of Southern Mississippi, Gulf Coast Research Laboratory Campus, Ocean Springs, MS 39564, USA

**Keywords:** Tanaidomorpha, *Paratanais*, new species, Caribbean, Puerto Rico

## Abstract

*Paratanais rosadi* **sp. n.** described from Puerto Rican coastal waters represents the first species of the genus from the northwestern Atlantic. It is distinguished from the other *Paratanais* species by a combination of characters, including article-2 of the maxilliped palp with a geniculate, finely-serrulate seta on inner margin; chela with stiff, geniculate, seta arising from propodus between fixed finger and dactylus and with short, stout, finely serrulate, seta on inner distal face of propodus adjacent to base of dactylus; carpus of pereopods 4−6 having three, instead of four stout modified spiniform setae distally, uropodal exopod distinctly shorter than endopodal article-1; and uropodal endopod with articles of about of equal in length. A key for the separation of *Paratanais* species from the Atlantic Ocean is presented.

## Introduction

Morales-Núñez (2011) have summarized the information on the crustacean order Tanaidacea presently known from Puerto Rico. They recorded a species belonging to the genus *Paratanais* Dana, 1852 from the waters off Culebra Island. Subsequently, additional specimens of this species were collected from La Parguera on the southwestern coast of Puerto Rico. After further examination it was determined that it represented and undescribed species. The description of this species is the subject of this paper.

The status of genus *Paratanais* was recently partially reviewed by Bird and Bamber (2013). Based on differences in the setation of the maxilliped, chela, and pereopods, they transferred five species, (i.e. *Paratanais denticulatus* Gutu & Ramos, 1995; *Paratanais intermedius* Dojiri & Sieg, 1997; *Paratanais malignus* Larsen, 2001; *Paratanais spinanotandus* Sieg, 1981; and *Paratanais vicentetis* Larsen, Nagaoka & Froufe, 2012) to the new genus, *Aparatanais* Bird & Bamber, 2013. Further they described four new species of *Paratanais* (i.e. *Paratanais caterae* Bird & Bamber, 2013; *Paratanais hamulus* Bird & Bamber, 2013; *Paratanais incomptus* Bird & Bamber, 2013; and *Paratanais puia* Bird & Bamber, 2013) from New Zealand waters. *Paratanais coelhoi* Araújo-Silva & Larsen, 2012 described was not treated by Bird and Bamber (2013).

Twenty-seven nominal species are currently attributed to the genus *Paratanais* (Anderson 2013; WoRMS). At present, seven species have been reported from the Atlantic Ocean. Three of these species *Paratanais hessleri* Kudinova-Pasternak, 1985; *Paratanais martinsi* Bamber & Costa, 2009; and *Paratanais pseudomartinsi* Larsen, 2012, are known from their respective type localities in the NE Atlantic, and a single species, *Paratanais elongatus* (Dana, 1849) is reported from the NW Atlantic (Bamber and Costa 2009, Larsen 2012, Kudinova-Pasternak 1985; Makkaveeva 1968). Three of these species are reported from the South Atlantic, *Paratanais euelpis* Barnard, 1920 from the SE Atlantic and two, *Paratanais coelhoi* Araújo-Silva & Larsen, 2012 and *Paratanais oculatus* Vanhöffen, 1914, from the SW Atlantic (Araújo-Silva and Larsen 2012, Barnard 1920, Silva-Brum 1973).

Of the three NE Atlantic species, *Paratanais hessleri* is the most poorly known. Its type material is based on single incompletely described and illustrated female specimen collected on the slope of the Great Meteor Seamount. The other SE Atlantic species, *Paratanais martinsi* and *Paratanais pseudomartinsi*, which were both described from the Azores, are quite similar morphologically (Larsen 2012).

Makkaveeva (1968) reported *Paratanais elongatus* as “*Leptochelia [=Paratanais] elongatus* (Dana, 1849)” from Cuban waters; however, Bamber (1998) suggested that her “poorly figured” specimens might have more affinities with the South African *Paratanais euelpis*. Additionally, Heard et al. (2004) reported “*Paratanais* sp. A” from Florida waters including Port Everglades, Florida Keys (Long Key), and Florida West Coast (Tampa Bay) and they suggested that the Cuban specimens reported by Makkaveeva might be conspecific with the one found by them in South Florida material.

From the equatorial Southwest Atlantic, Silva-Brum (1973) reported *Paratanais oculatus* (Vanhöffen, 1914) from Brazilian coastal waters; this species was previously known from Sub-Antarctic waters (Vanhöffen 1914; Shiino 1978). Silva-Brum’s identification, however, was questioned by Araújo-Silva and Larsen (2012), who described *Paratanais coelhoi* from the same general locality. The remaining South Atlantic species, *Paratanais euelpis*, was briefly described from the Southwest coast of South Africa (Barnard 1920) and later redescribed in detail by Lang (1973).

## Materials and methods

**Figure 1.**
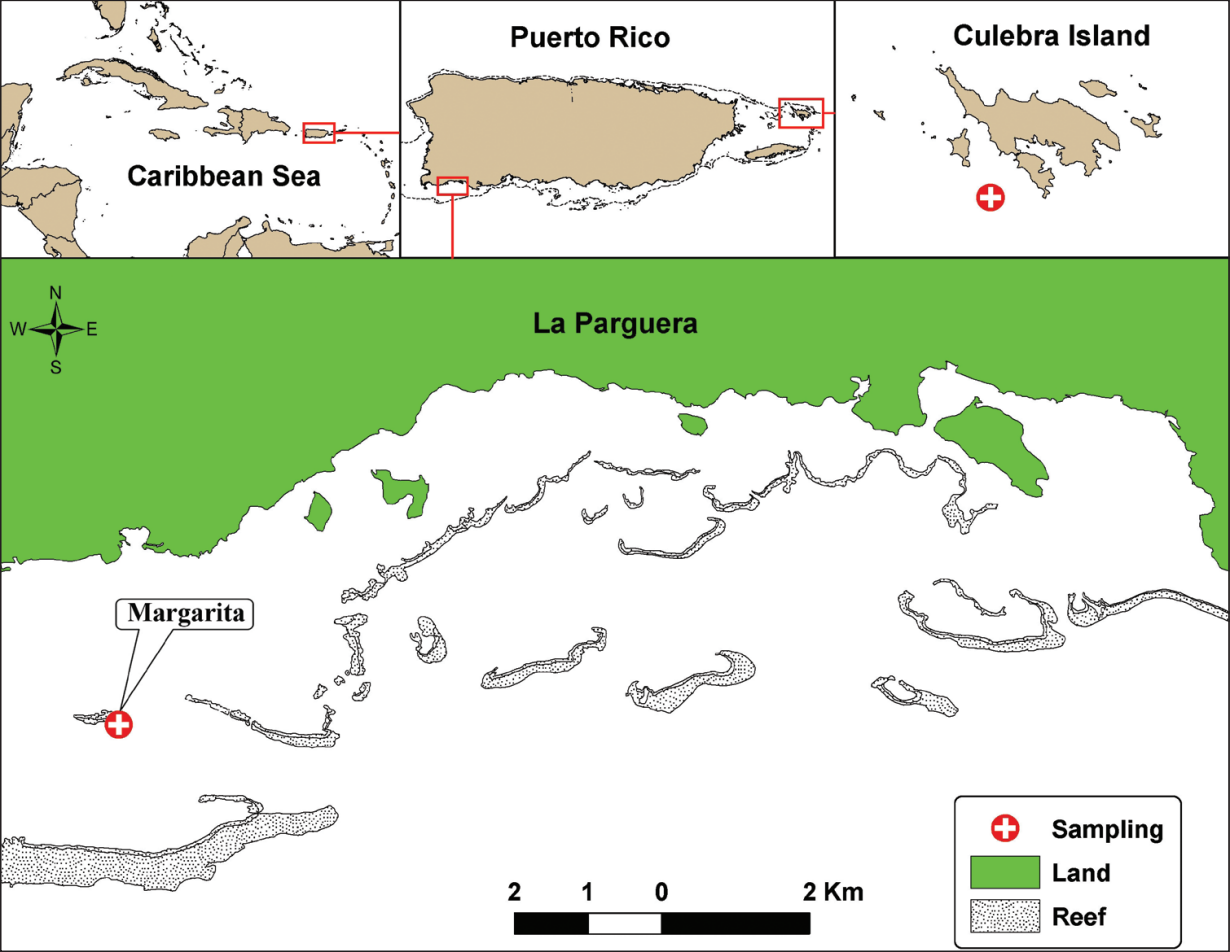
Geographic location of La Parguera, Southwest Puerto Rico, and Culebra Island, Eastern Puerto Rico, indicating the sampling stations where *Paratanais rosadi* sp. n., were found.

Part of the material was diver-collected during 2002 using PVC corers at a depth of 28 m off Culebra Island (eastern Puerto Rico). During 2008 larger series of specimens, including the type material was collected with a benthic grab at a depth of 14.9 m near Margarita Key in the southwestern region of La Parguera Natural Reserve (Fig. 1). Samples were processed using the methods described by Morales-Núñez and Kornicker (2007) and Morales-Núñez et al. (2010).

Type material has been deposited in the National Museum of Natural History, Smithsonian Institution, Washington DC, (USNM), and the Gulf Coast Research Laboratory Museum, Ocean Springs, Mississippi MS, (GCRL). All measurements are in millimetres (mm). Total body length (TL) is measured from the tip of the rostrum to the end or tip of the telson. The terminology used in this paper, unless otherwise stated, follows that of Larsen (2003). Abbreviations: TL = total length; millimetres = mm.

## Systematics

### Order Tanaidacea Dana, 1849
Suborder Tanaidomorpha Sieg, 1980
Superfamily Paratanaoidea Lang, 1949
Family Paratanaidae Lang, 1949
Subfamily Paratanainae Lang 1949

#### Genus Paratanais

Dana, 1852

http://species-id.net/wiki/Paratanais

##### Type species.

*Paratanais elongatus* Dana, 1849 (see Bamber 1998).

##### Generic diagnosis.

See Bird and Bamber (2013).

#### Paratanais rosadi sp. n.

http://zoobank.org/D0D83D3C-8FA3-4378-9A91-5FE2D8B1E77B

http://species-id.net/wiki/Paratanais_rosadi

**Figure 2.**
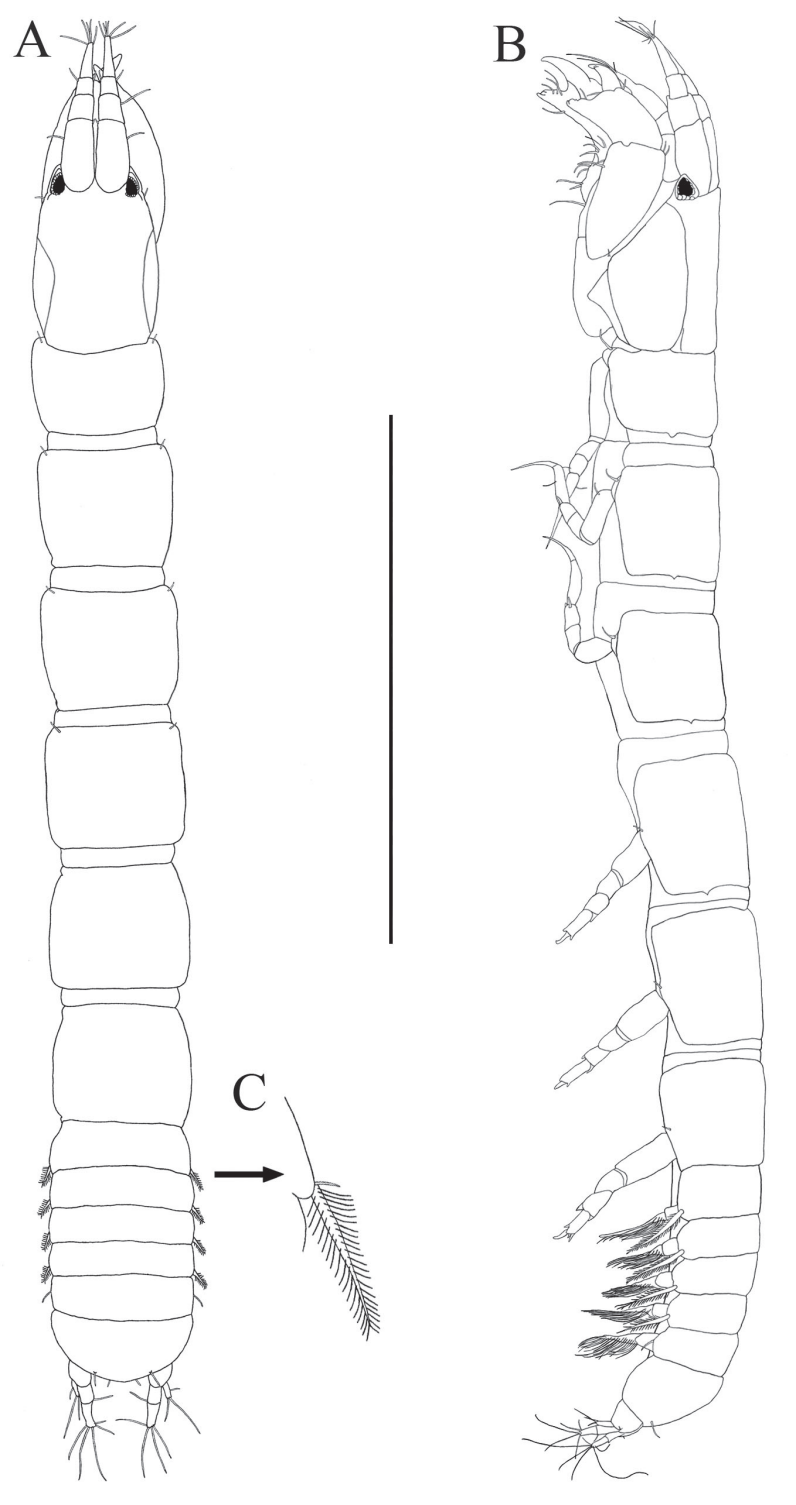
*Paratanais rosadi* sp. n., holotype female: **A** dorsal view **B** lateral view **C** enlargement of articulated setulate seta on pleonite-1. Scale bar **A**–**B** 1.0 mm.

Figs 2 [Fig F3] [Fig F4] [Fig F5] [Fig F6] [Fig F7] [Fig F8] –9 ; 10C, G, J, M and P

##### Material examined.

*Holotype*: adult female (USNM 1231351), 17°55'57.70"N, 67°06'53.36"W, Margarita Southwest of La Parguera, Puerto Rico, depth 14.9 m, collected on August 1 of 2008. *Paratypes*: one male (USNM 1231352), two females (USNM 1231353); two females, (GCRL 6529), the same collection data as for holotype.

Additional specimens from the type locality are in the collection of the authors.

##### Diagnosis.

**Female.** Pleon shorter than pereonites 5−6 combined. Antennule with cap-like terminal article. Antenna article-2 with length twice depth in lateral aspect, ventral marginal sub-linear, lacking shallow apophysis, small simple seta subdistally. Maxilliped palp article-2 with inner margin bearing geniculate, finely-serrulate, seta. Chela with propodus having geniculate, narrow, stiff, seta arising at inner base of fixed finger adjacent articulation with dactylus and extending distally: inner face with small, short, stout, finely serrulate seta on inner distal face of propodus adjacent to base of dactylus. Pereopods 4−6 having carpus distally with three modified, stout spiniform setae and small simple seta. Uropodal exopod uniarticulate, length about twice width, and shorter than endopodal article-1; endopod with both articles about same length. **Male.** Small, length about 1.2 mm. Carapace length about equal to that of first three pereonites combined; eyes large with diameter about half length of carapace. Pereonites 4−5 slightly longer than pereonites 1−3, and 6. Pleonites as long as pereonites 2−5 combined length. Antennule peduncle article about 1.3 times as long as wide; antennular flagellum with four-articles, without detectable terminal cap-like article; flagellum article-2 shorter than articles 3−4 combined. Uropod endopod bi-articulated.

##### Etymology.

This species is named in honour of Marcos Rosado Ruiz who has instrumental in assisting the senior author in collection of the specimens used in this study.

##### Description - adult female.

*Body* (Fig. 2A). Length about 2.8 mm, about 12.5 times width.

*Carapace* (Fig. 2A). About 15% TL and about twice length of pereonite-1. Ocular lobes with eyes, visual elements present, with demarked lines on carapace indicating possible union of lateral plates.

*Pereon* (Fig. 2A). About 65% of TL, pereonite-1 shorter than other pereonites; pereonites 2−6 subequal in length; all pereonites subrectangular; all pereonites wider than long.

*Pleon* (Figs 2A‒C). About 15% TL, shorter than pereonites 5−6 combined; pleonites-1 and -5 of nearly equal lengths, distinctly wider than long, slightly larger than others pleonites; pleonites 2−4 subequal; pleonites 1−4 each with pair of swollen lateral, setulose setae (Fig. 2C); pleonite-5 with lateral pair of short simple setae (Fig. 2B).

*Pleotelson* (Figs 2A, 4F). About 5% TL, longer than pleonite-5, with four simple setae distally.

**Figure 3. F3:**
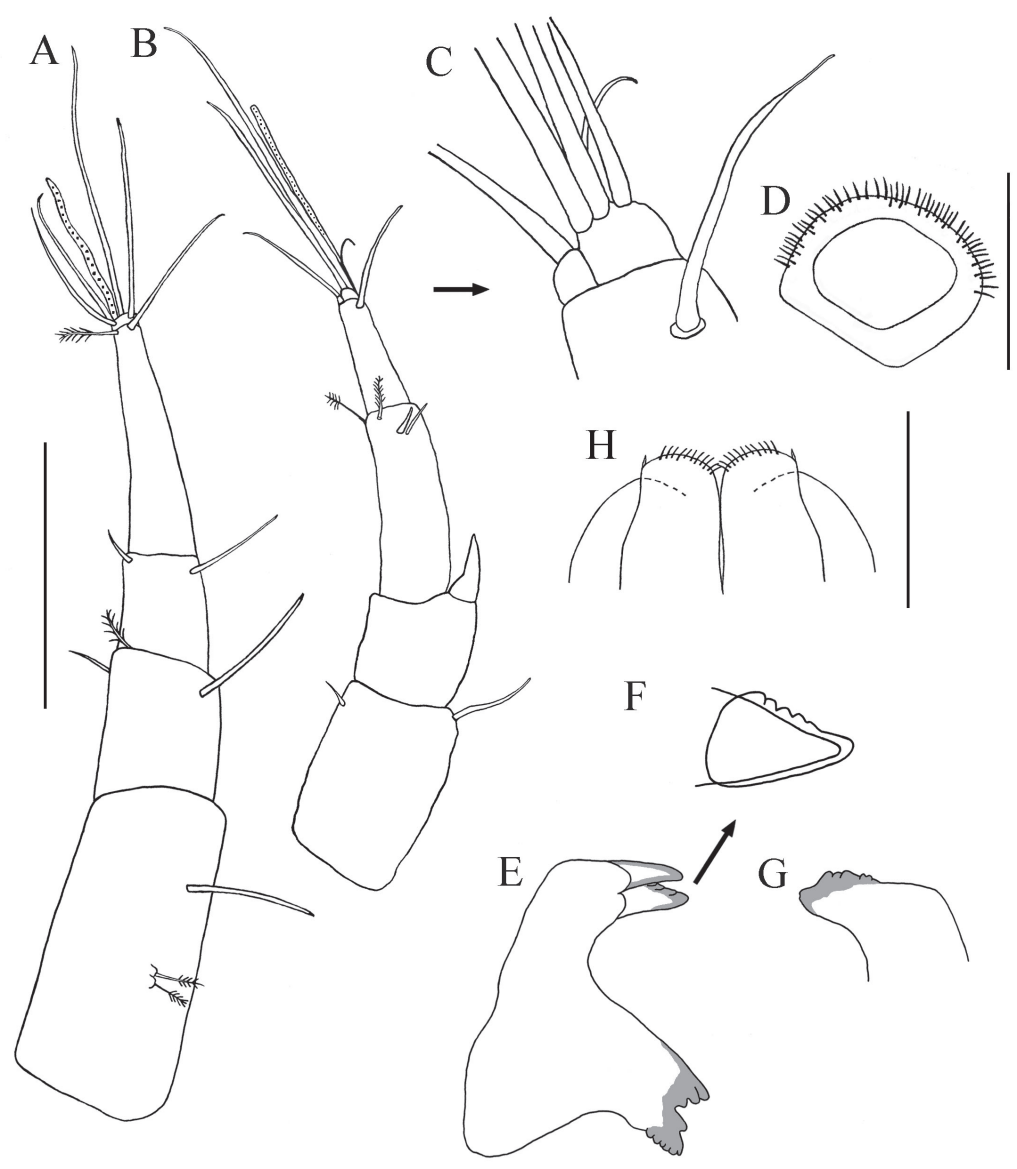
*Paratanais rosadi* sp. n., holotype female: **A** antennule, lateral view **B** antenna, lateral view **C** enlargement of tip of the antenna **D** labrum **E** left mandible **F** lacinia mobilis **G** right mandible **H** labium. Scale bar **A**–**G** 0.1 mm.

*Antennule* (Fig. 3A). Slightly shorter than carapace. Article-1 length about 2.4 times width, two broom-setae laterally on mid-dorsal margin and simple seta distally on mid-dorsal margin. Article-2 length about third that of article-1, one simple seta on dorsodistal margin, and one simple and one broom-seta disto-ventrally. Article-3 with length about 1.5 times width, dorsal and ventral seta on distal margin. Article-4 elongate, equal length of article 2−3 combined, distally with broom-seta and long simple seta. Small, terminal, cap-article with aesthetasc and four simple setae of varying length.

*Antenna* (Figs 3B‒C). Article-1 greatly reduced (not illustrated). Article-2 length about 1.4 times depth, ventral margin sub-linear with small simple seta subdistally; dorsal margin with long simple seta distally. Article-3 slightly wider than long, with strongly developed, stout disto-dorsal spiniform seta. Article-4 about three times width, with two short simple setae on middle-distal margin and two broom-seta on distoventral margin. Article-5 with disto-dorsal simple seta. Article-6 minute, with one articulated cluster of one small curved seta and three long setae, and single articulated seta (Fig. 3C).

**Figure 4. F4:**
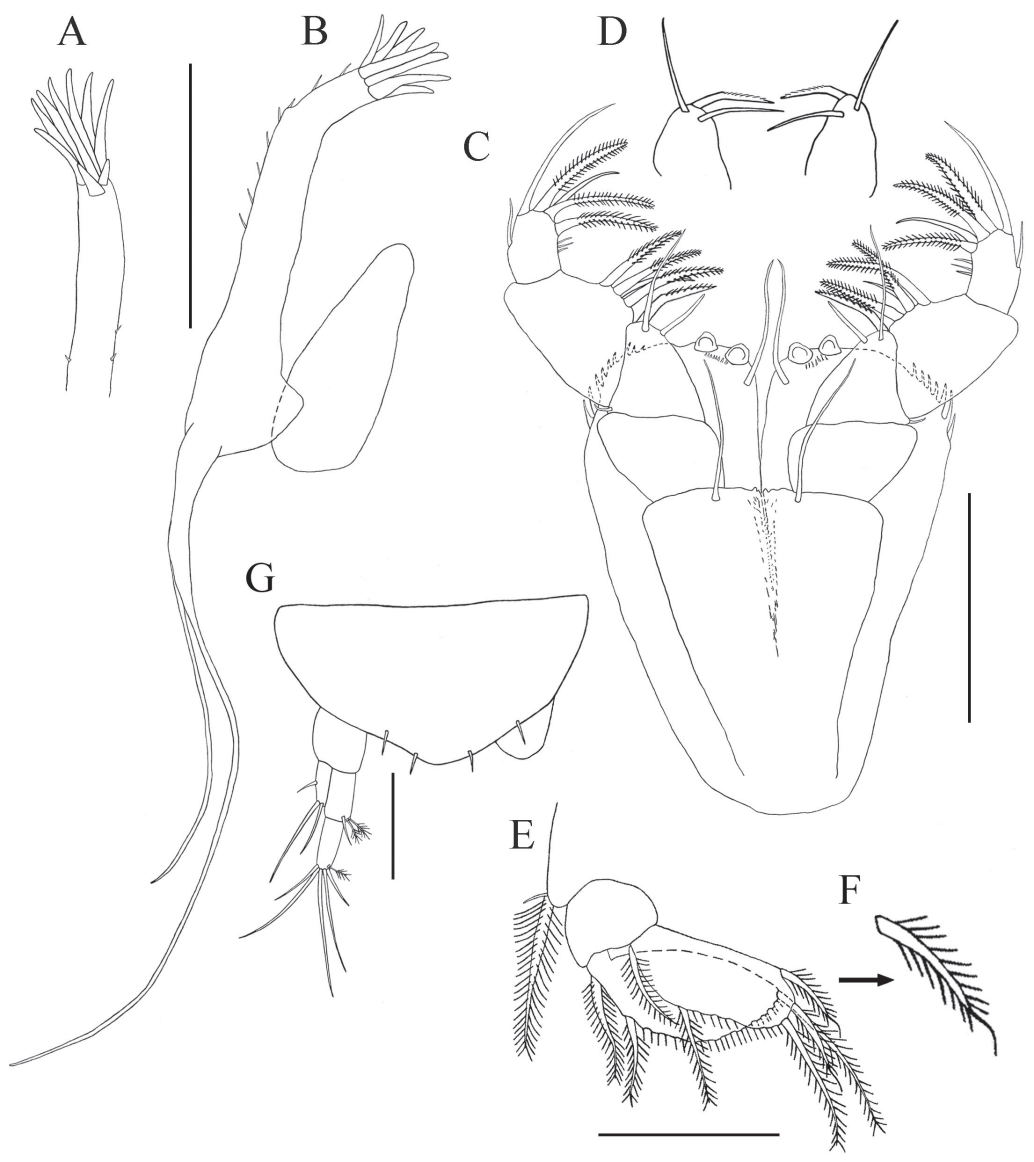
*Paratanais rosadi* sp. n., holotype female: **A** maxillule **B** maxillule and maxilla **C** maxilliped **D** enlargement of palp article-2 **E** pleopod **F** enlargement of plumose seta, with whip-like tip **G** uropod. Scale bar **A**–**F** 0.1 mm.

*Mouthparts*. *Labrum* (Fig. 3D). Hood-shaped, distal edge finely setose. *Mandibles* (Figs 3E‒G): *molar process* well developed; left mandible with smooth, strong incisor without crenulate upper margin, *lacinia mobilis* sub-triangular with four to five shallow subdistal denticles (Figs 3E–F); right mandible (Fig. 3G) with strong crenulate incisor and weakly bifid tip. *Labium* (Fig. 3H): with two lobes, inner lobe finely setose distally, with minute spiniform seta on outer distal margin. *Maxillule* (Figs 4A‒B): endite with nine distal spiniform setae, outer margin with short simple setae; palp with two long terminal setae of unequal length (Fig. 4B). *Maxilla* (Fig. 4B): subovally elongate.

*Maxilliped* (Figs 4C‒D). Basis fused, long seta near articulation with palp extending distally to or near distal margin of endites; endites fused medially in proximal third, inner lobes with distal margin bearing seta and two medial flat tubercles, inner lobes serrate on outer-distal margin. *Palp*: article-1 naked; article-2 triangular with simple seta on outer proximal margin, inner margin with two simple setae and geniculate, finely-pectinate (visible at magnification 100×), spiniform seta (Fig. 4D); article-3 with three setulose setae on inner margin; article-4 with four (three setulose and one simple) setae on distal margin, simple seta on outer margin, and two or three simple setae on inner margin. *Epignath*: not recovered.

**Figure 5. F5:**
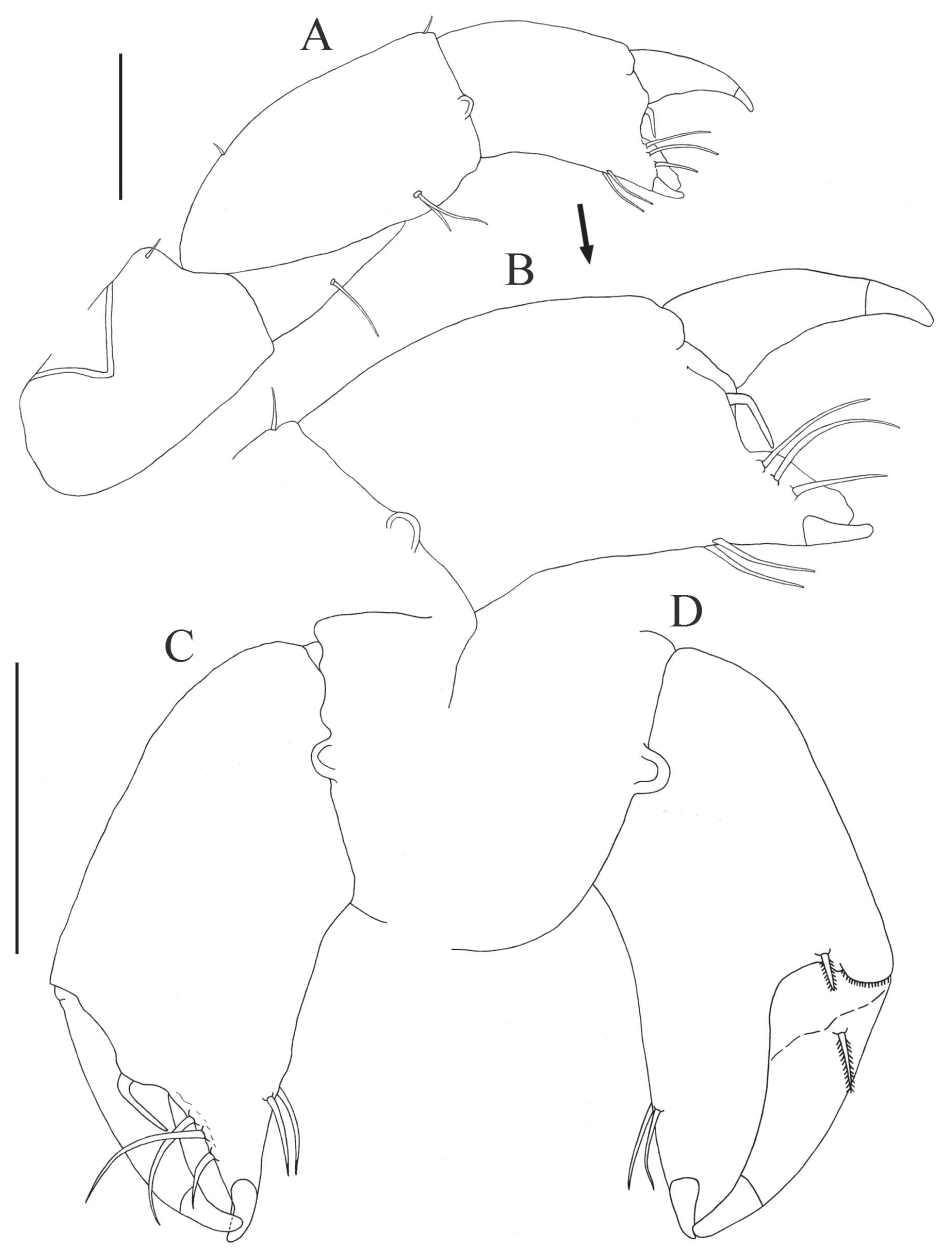
*Paratanais rosadi* sp. n., holotype female: **A** right cheliped, lateral view **B** enlargement of propodus and dactylus of right cheliped, lateral view **C** propodus and dactylus of left cheliped, lateral view **D** propodus and dactylus of left cheliped, inner view. Scale bar **A**, **C** and **D** 0.1mm.

*Cheliped lateral aspect* (Figs 5A–C). Sclerite sub-triangular, dorsally inserted, naked. Basis length about 2.1 times width, simple seta on disto-dorsal margin. Merus triangular, with simple seta on mid-ventral margin. Carpus length 1.8 times width, with two (one proximal and one distal) short simple setae on dorsal margin, and two ventral simple setae on subdistal, ventral margin. Chela (*lateral aspect*) (Figs 5A−C): propodus length 1.4 times width; fixed finger with two ventral seta and three simple setae on incisive margin adjacent; stiff, geniculate, seta arising from propodus between fixed finger and dactylus, extending distally between fixed finger and dactylus. Dactylus slightly longer than fixed finger, curved distally, unguis not fused. Chela (*Inner aspect*) (Fig. 5D): Propodus with short setulate seta distally near articulation of dactylus. Dactylus with short setulate seta proximally on inner sub-dorsal margin.

**Figure 6. F6:**
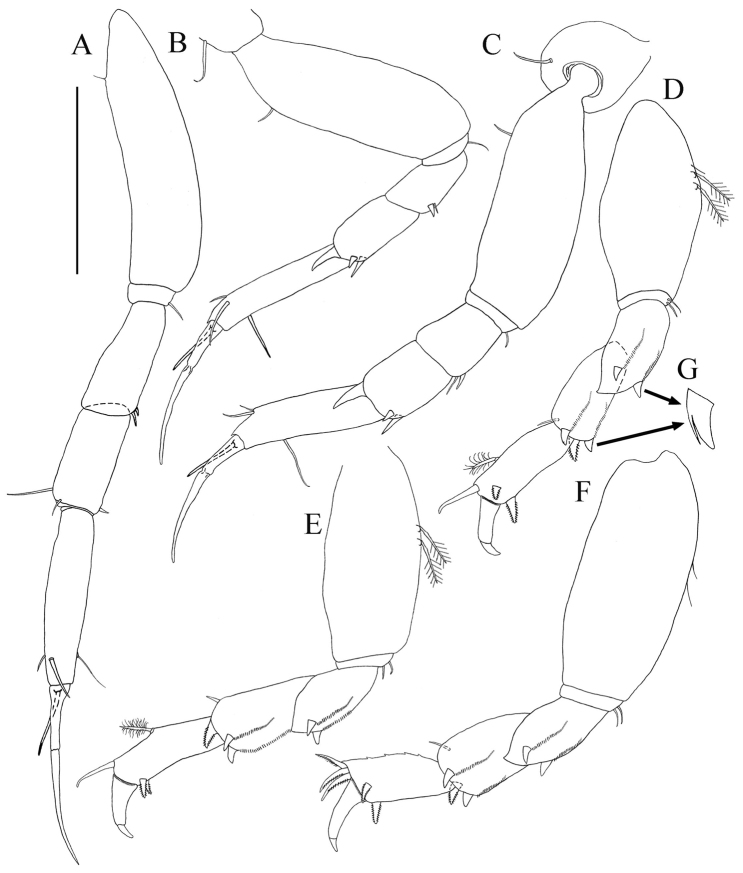
*Paratanais rosadi* sp. n., holotype female: **A** pereopod 1 **B** pereopod 2 **C** pereopod 3 **D** pereopod 4 **E** pereopod 5 **F** pereopod 6 **G** enlargement of bifurcate spiniform setae. Scale bar **A**–**F** 0.1 mm.

*Pereopod-1* (Figs 2B, 6A). Longer than other five pereopods. Coxa with simple seta (Fig. 2B). Basis slender, length about 3.8 times width, with dorso-proximal simple seta. Ischium about three times width with single simple ventral seta. Merus length about 2.1 times width, with two ventro-distal small simple setae. Carpus with length about 2.1 times width, one short and one long disto-dorsal simple setae and one ventro-distal simple seta. Propodus with length about 4.5 times width, with two sub-distal simple setae on dorsal margin and one sub-distal seta ventrally. Dactylus and unguis combined about as long as propodus, with simple proximal seta, unguis longer than dactylus.

*Pereopod-2* (Figs 2B, 6B). Coxa with simple seta (Fig. 2B). Basis length about 2.7 times width, with supraproximal seta. Ischium length about 3.0 times width, with simple ventral seta. Merus length about 1.5 times width, with simple seta and one spiniform seta, disto-ventrally. Carpus length about 1.8 times width, with one longer disto-dorsal spiniform seta and two disto-ventral spiniform setae. Propodus length about 5.1 times width, with two sub-distal simple setae on dorsal margin and one sub-distal simple seta ventrally. Dactylus and unguis together longer than propodus and not fused, with simple proximal seta, unguis longer than dactylus.

*Pereopod-3* (Fig. 6C). Similar to pereopod 2, except basis longer. Merus, carpus and propodus wider.

*Pereopod-4* (Figs 6D, G). Basis stout, length about twice width, with simple seta and two broom setae on mid-ventral margin. Ischium length about 6.5 times width, with two ventral simple setae. Merus length about 1.9 times width, with two short asymmetrical bifurcate spiniform setae on disto-ventral margin (Fig. 6G), and row of setules on distal half of ventral margin. Carpus length about 1.5 times width, distally with simple disto-ventral seta, and three stout modified spiniform setae (two asymmetrical bifurcate and one bipinnate spiniform seta), with row of setules on distal half of ventral margin. Propodus length about 2.6 times width, with mid-dorsal broom seta and disto-dorsal spiniform seta, with two bipinnate spiniform setae on distoventral margin, with distal row of setules. Dactylus and unguis claw-like, together almost half length of propodus, dactylus longer than unguis, curved and not fused.

*Pereopod-5* (Fig. 6E): Similar to pereopod-4, except basis without simple setae on mid-ventral margin.

*Pereopod-6* (Fig. 6F): Similar to pereopod-5, except basis length about 2.3 times width with two simple setae on mid-ventral margin. Propodus with four short spines on dorsal margin and without mid-dorsal broom seta, with three pectinate distal spiniform setae. Dactylus and unguis together about 1/3 length of propodus.

*Pleopod* (Figs 4E−F). Five similar, well-developed, biramous pairs. Basal article broad, naked. Rami lengths slightly more than twice width; proximal plumose seta on distal inner margin adjacent to articulation with basis. Endopod with inner and distal margins bearing ten long plumose setae, distal most seta modified with whip-like tip (Fig. 4F); sub-distal lateral margin with seta modified with whip-like tip. Exopod with inner and distal margins bearing about 15 long plumose setae, outer margin naked.

*Uropod* (Fig. 4G). Biramous, basis naked. Exopod uniarticulate, shorter than endopod article-1, with simple seta on mid-outer margin, and two simple setae (outer longest) on distal margin. Endopod biarticulated, article-1 as long as peduncle, with one simple and two broom setae on inner distal margin; article-2 length about subequal to length of article-1, with five simple and one broom setae on distal margin.

##### Description - adult male.

**Figure 7. F7:**
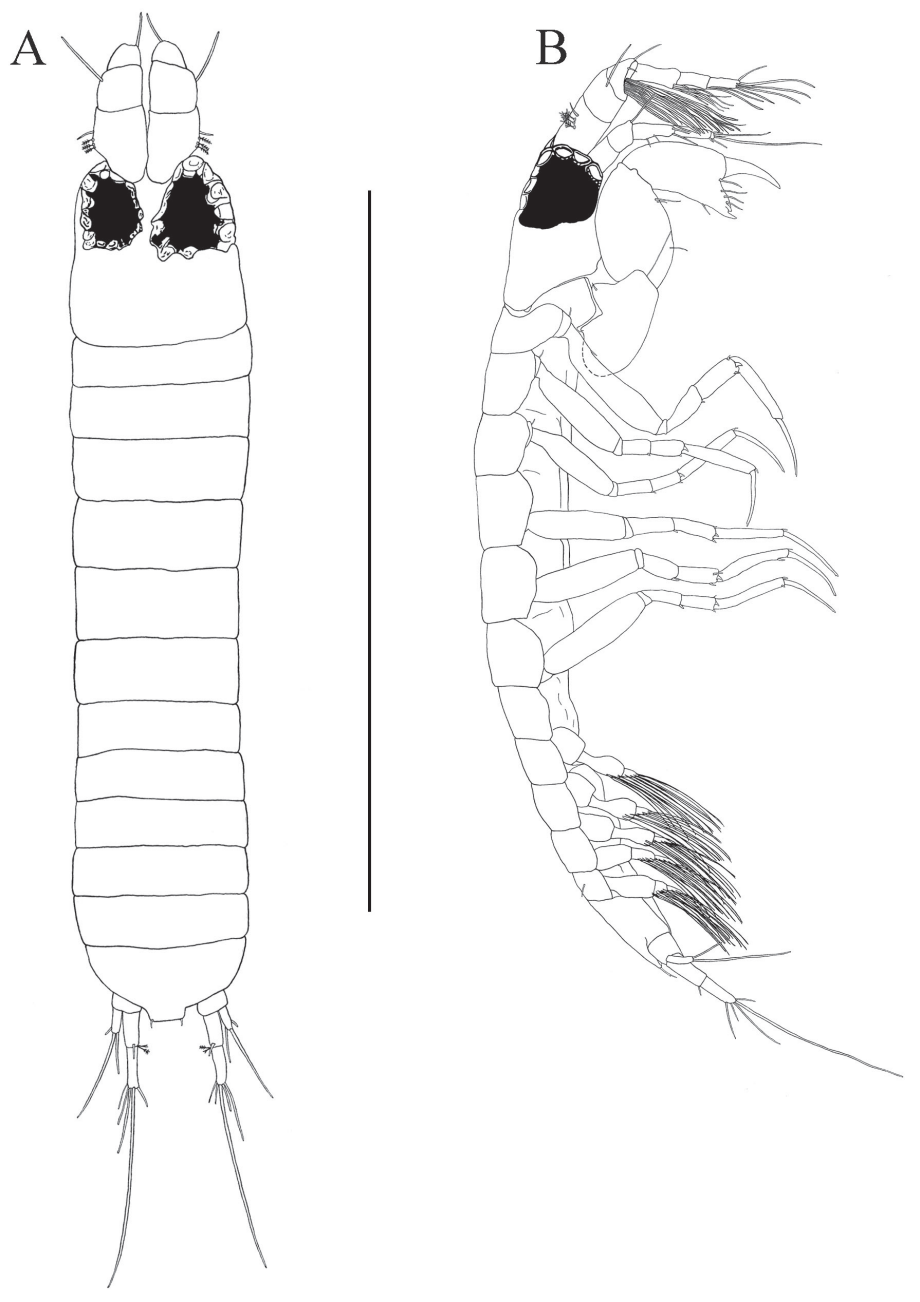
*Paratanais rosadi* sp. n., paratype male: **A** dorsal view **B** lateral view. Scale bar **A**–**B** 1.0 mm.

*Body* (Figs 7A–B). Length about 1.20 mm, about 4.8 times as long as wide, smaller than female.

*Carapace* (Fig. 7A). About 20% TL, nearly as longer as wide; quadrate; naked; as long as pereonites 1 to 3 combined; ocular lobes bearing large darkly pigmented, multifaceted eyes, diameter about half length of carapace and about eight times that of female eye.

*Pereon* (Fig. 7A). Slightly over 40% TL, pereonites sub-rectangular, and wider than long; pereonites 1 to 4 progressively longer; pereonite-5 about equal in length to pereonite-4; pereonite-6 shorter.

*Pleon* (Fig. 7A). About 30 % TL, as long as pereonite 2−5 combined; all pleonites subequal.

**Figure 8. F8:**
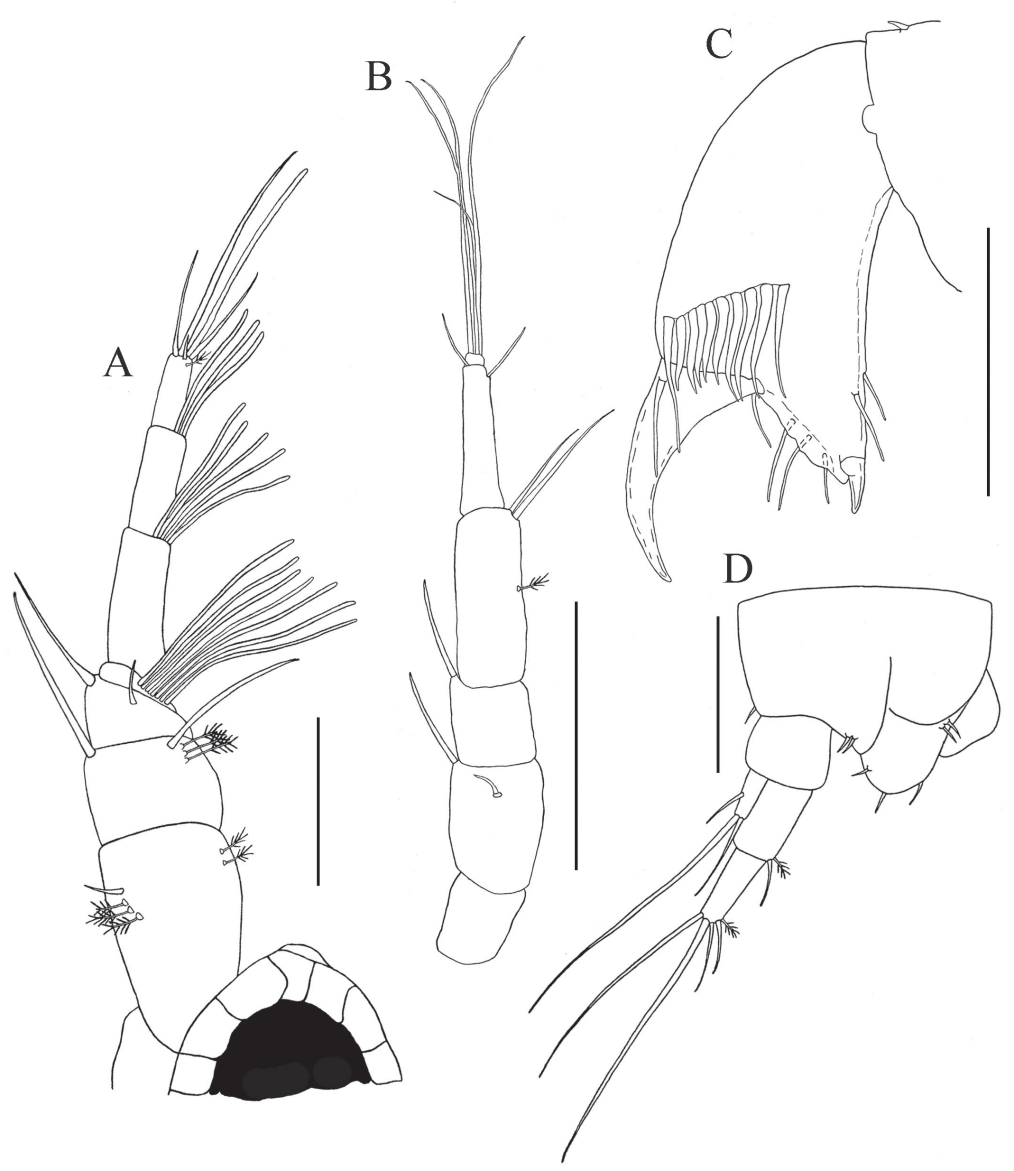
*Paratanais rosadi* sp. n., paratype male: **A** antennule, lateral view **B** antenna, lateral view **C** propodus and dactylus of right cheliped, inner view **D** uropod. Scale bar **A**‒**D** 0.1 mm.

*Pleotelson* (Fig. 7A). Little less than 10% TL, length about 2.1 times width; subequal length to pleonite-5, with two small apical simple setae on each side;

*Antennule* (Fig. 8A). With seven articles. Article-1 length about 1.3 times width, with four setae (1 simple and 3 broom) close to the middle of dorso-lateral margin, and with two broom setae on ventro-lateral subdistal margin. Article-2 length about 1.5 times width, with long disto-dorsal simple seta, and one simple and three broom on ventro-lateral distal margin. Article-3 with long disto-dorsal simple seta and small simple lateral seta. Article-4 with dense proximal group of aesthetasc ventrally. Article-5 length about 2.5 times width and with disto-ventral row of aesthetasc. Article-6 length about 2.7 times width, with disto-ventral row of aesthetascs. Article-7 length about 2.8 times width, with one long, one small, one broom setae and one aesthetasc, distally.

*Antenna* (Fig. 8B). Article-1 length about 1.1 times width. Article-2 length about 1.4 times width, with large disto-dorsal simple seta and simple seta on sub-distal lateral margin. Article-3 little wider than long, with large disto-dorsal simple seta. Article-4 length about 2.6 times width, with broom seta on mid-ventral margin and two long simple setae on disto-ventral margin. Article-5 elongate about 5.0 times as long as wide, with dorso-distal simple seta and simple seta on sub-distal ventral margin; article-6 tiny and with four (three long and one short) simple setae.

*Cheliped lateral aspect* (Figs 7B, 8C). Slightly longer than that of the female. Basis length about twice width. Carpus length 1.5 times without short simple setae on dorsal margin. Propodus with length 1.6 times width. *Inner face* (Fig. 8C). Propodus 1.6 times as long as wide; with inner face having “comb row” of ten stout setae just proximal to articulation with dactylus (movable finger); fixed finger with strong spine distally, two simple setae ventrally, three simple setae on outer incisive margin, and single simple seta near articulation of dactylus. Dactylus longer than fixed finger, distally curved and unfused; single dorso-proximal simple seta on inner margin.

**Figure 9.**
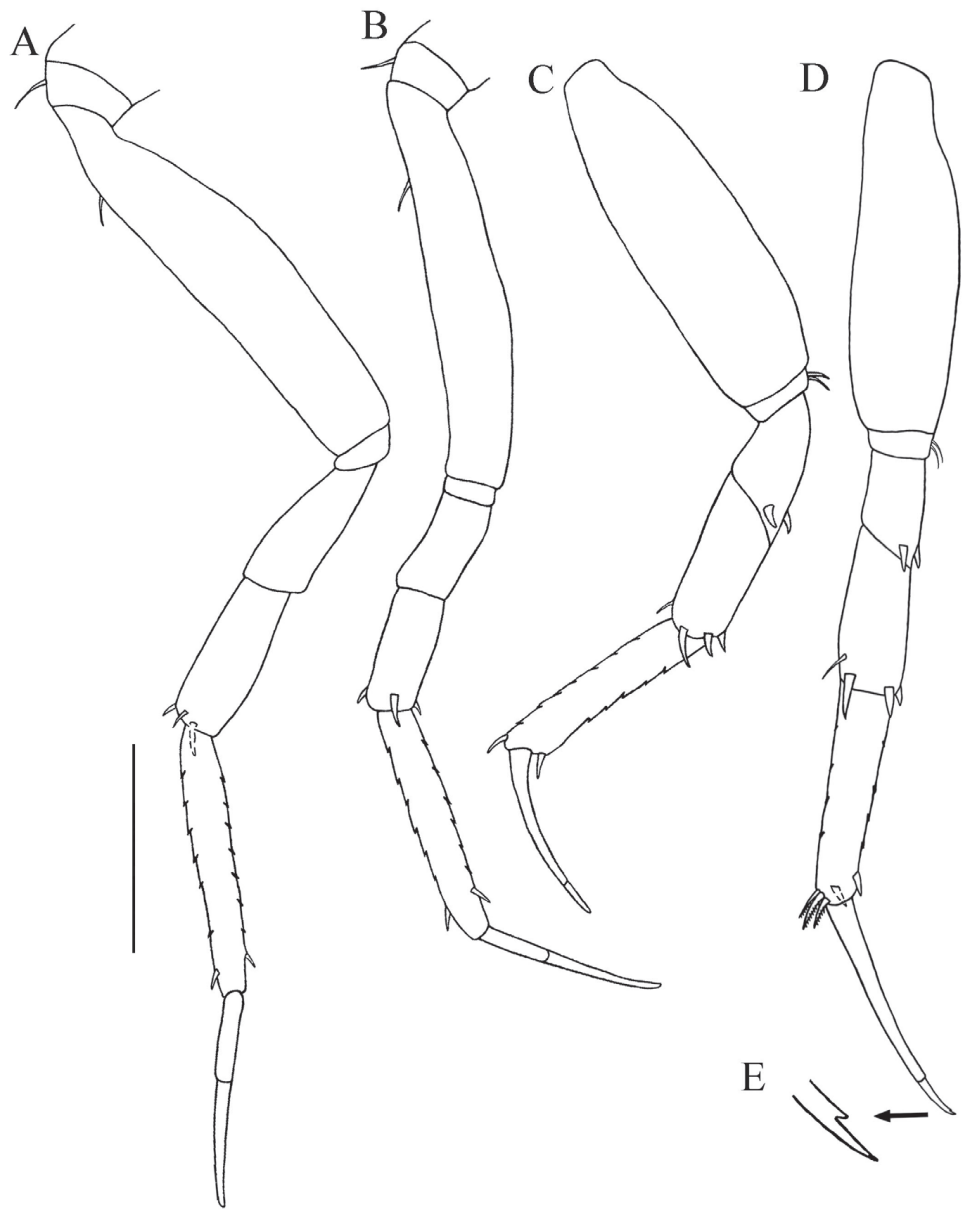
*Paratanais rosadi* sp. n., paratype male: **A** pereopod 1 **B** pereopod 2 **C** pereopod 4 **D** pereopod 6 **E** enlargement of tip of the unguis. Scale bar **A**‒**D** 0.1 mm.

*Pereopod-1* (Fig. 9A). Longer than other pereopods. Similar to that of female, except for basis, carpus, and propodus longer. Ischium naked. Propodus with dorsal and ventral margin crenulate. Dactylus and unguis combined shorter than propodus; unguis slightly longer than dactylus.

*Pereopod-2* (Fig. 9B). Similar to pereopod-1, except for merus, carpus, propodus, and dactylus shorter.

*Pereopod-3* (not figured). Similar to pereopod-2

*Pereopod-4* (Fig. 9C). Basis a little wider than in pereopods 2–3, appearing naked, length about 3.2 width. Ischium length about 3.0 times width, with two ventral simple setae. Merus length about 1.9 times width, with two short spiniform setae, disto-ventrally. Carpus length about 2.3 times width, with simple disto-dorsal seta and three spiniform setae. Propodus length 3.3 times width, with one spiniform seta on dorsal and ventral distal margin, with dorsal and ventral margin crenulate. Dactylus and unguis combined shorter than propodus; dactylus much longer than unguis, not fused.

*Pereopod-5* (not figured). Similar to pereopod–4.

*Pereopod-6* (Figs 9D–E). Similar to pereopod-4, except for propodus with three pectinate distal spiniform setae. Dactylus and unguis together longer than propodus, dactylus longer than unguis, tip of unguis bifid (Fig. 9E).

*Pleopod* (not figured). Five similar, but more strongly developed than in female with longer natatory setae. Endopod with inner and distal margins bearing eleven long plumose setae, distal most seta modified with whip-like tip; sub-distal lateral margin with seta modified with whip-like tip. Exopod with inner and distal margins bearing about 12 long plumose setae, outer margin naked.

*Uropod* (Fig. 8D). Similar to female, with minor qualitative differences in setation and all simple setae are longer than female.

##### Distribution.

Currently know only from the type locality, at the depths from 15 to 28 m, on sandy substrata.

##### Remarks.

*Paratanais rosadi* sp. n., can be distinguished from the other previously described species by having carpus with only three distinct, modified, stout seta on pereopods 4−6; all others species have four distinct carpal spines. The female of *Paratanais rosadi*, appears most similar to the northeastern Atlantic species, *Paratanais martinsi* from the Azores and *Paratanais euelpis* from South African; *Paratanais rosadi* can be distinguished from *Paratanais martinsi* by (1) lacking a distinctly buttressed seta on antenna article-2; (2) having antennal article-2 with small simple seta on subdistal-ventral margin, not arising from apophysis or process; and (3) having uropodal endopod article-1 about same length as article-2. Owing to difficult in reliably distinguishing them, *Paratanais martinsi* and *Paratanais pseudomartinsi* are not separated in the Key to the Atlantic presented herein.

Based on Lang’s detailed redescription of *Paratanais euelpis*, *Paratanais rosadi* differs from that species by the presence of a single small, short, setulate seta on the inner face of chela at the articulation with the dactylus, by having the endopodal articles about the same length; and by the stout exopod about twice as long as wide and being shorter than endopodal article-1.

The new Puerto Rican species differs from the Brazilian species, *Paratanais coelhoi* by having (1) antennule article-1 being more elongate; (2) article-2 of the maxillipedal palp having inner margin with finely serrulate geniculate seta medially and two simple setae; and (3) merus of pereopod-2 with distinct spiniform seta on ventral margin.

Bamber and Costa (2009) considered *Paratanais hessleri* from the mid-North Atlantic as a species *incertae sedis*. Based on the Kudinova-Pasternak’s (1985) illustrations, the presence of a biarticulate exopod distinguishes that deep-water species from *Paratanais rosadi* and so from the other Atlantic species of *Paratanais*. Although the description by Kudinova-Pasternak does not give details for the setation on pleonites 1 to 4, her illustration indicates the presence of only a simple seta on their pleura. Since all other known members of the genus *Paratanais*, with the possible exception of *Paratanais oculatus* sensu Shiino (1978), have swollen setulate setae present, her illustration is most likely incorrect. Notwithstanding, re-examination of Kudinova-Pasternak’s holotype and Shiino’s specimens is needed to clarify their status.

Vanhoffen’s (1914) original description for *Paratanais oculatus* is brief and incomplete and his few illustrations lack detail. For the present we tentatively refer both *Paratanais hessleri* and *Paratanais oculatus* to the *Paratanais*. Based on Shiino’s (1978) description of *Paratanais oculatus* from Sub- Antarctic waters, the female of *Paratanais rosadi* can be distinguished from it by having (1) article-2 of the maxillipedal palp with finely serrulate and geniculate stiff seta and two simple setae (instead of four apparently simple setae) medially and (2) uropodal exopod composed of single article.

In our opinion, no meaningful comparisons can be made using the descriptions provided for *Paratanais oculatus* sensu Silva-Brum (1973) from Brazil and *Paratanais* (=*Leptochelia*) *elongatus* *sensu* Makkaveeva (1968) from Cuba. Until the original material for these records can be re-examined or additional material from the localities in question made available for study, their occurrence in the Atlantic Ocean will remain highly suspect.

**Figure 10.**
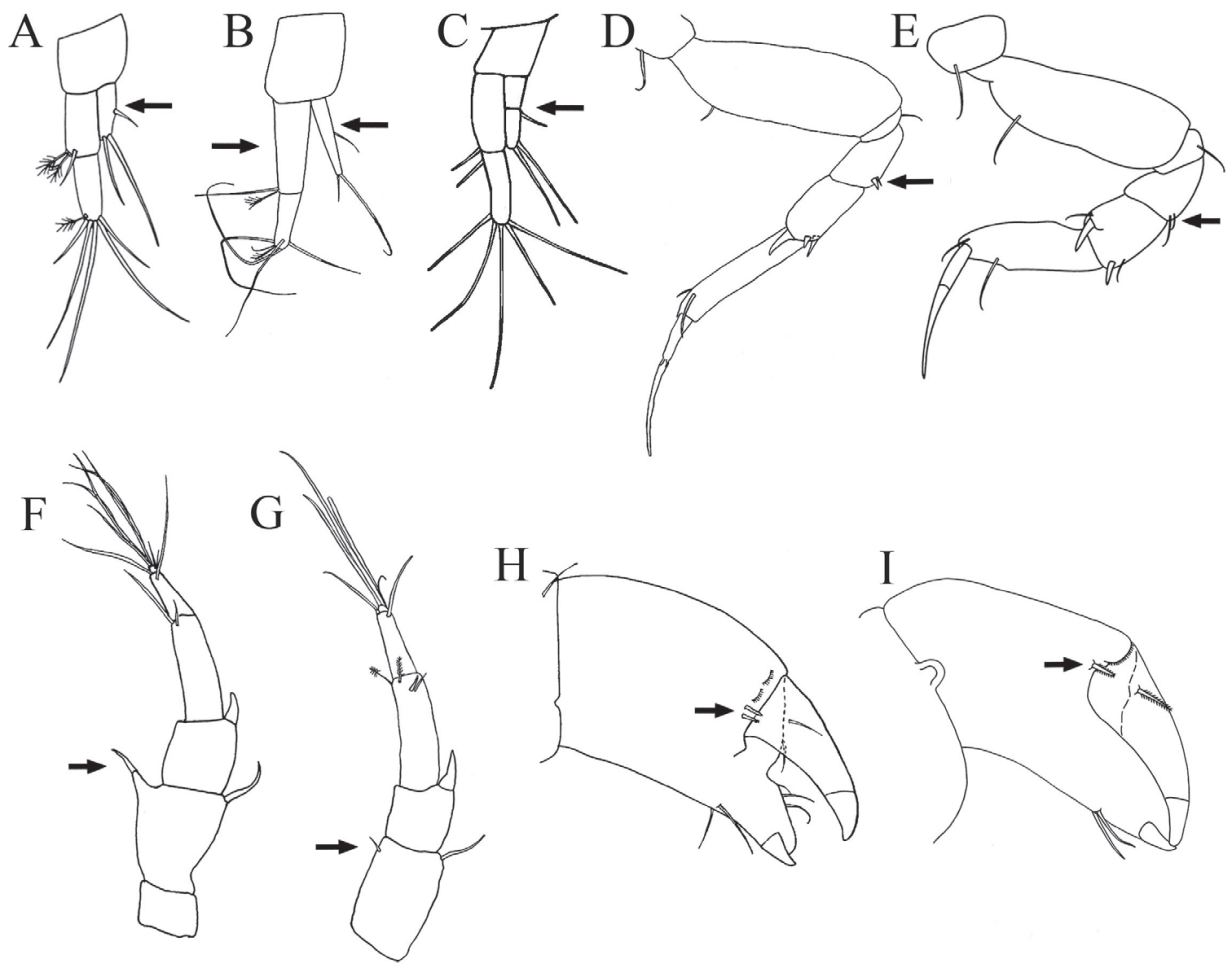
Uropods: **A** *Paratanais rosadi* sp. n. **B** *Paratanais euelpis* **C** *Paratanais hessleri*. Pereopod-2: **D** *Paratanais rosadi* sp. n. **E** *Paratanais coelhoi*. Antenna: **F** *Paratanais martinsi* **G** *Paratanais rosadi* sp. n. Chela: **H** *Paratanais euelpis* **I** *Paratanais rosadi* sp. n. [Figures modified from: this study (**A**, **D**, **G**, and **I**); Lang 1973 (**B** and **H**); Kudinova-Pasternak 1985 (**C**); Araújo-Silva & Larsen 2012 (**E**); Bamber & Costa 2009 (**F**)].

### Key to the currently recognized species of *Paratanais* known or reported from the Atlantic

**Table:** 

1	Uropod exopod uniarticulate (Figs 10A−B)	2
‒	Uropod exopod appearing biarticulate (Fig. 10C)	*Paratanais hessleri* Kudinova-Pasternak, 1985 [Northeast Atlantic: Great Meteor Seamount]
2	Pereopod-2 merus having spiniform seta on posterodistal margin (Fig. 10D)	3
–	Pereopod-2 merus lacking spiniform seta on posterodistal margin (Fig. 10E)	*Paratanais coelhoi* Araújo-Silva & Larsen, 2012 [Southwest Atlantic: Brazil]
3	Antennal article-2 with simple spiniform seta on distoventral margin arising from apophysis (Fig. 10F)	*Paratanais martinsi* Bamber & Costa, 2009 / *Paratanais pseudomartinsi* Larsen, 2012 [Northeast Atlantic: Azores]
–	Antennal article-2 with small simple, unbuttressed seta on subdistal-ventral margin, not arising from apophysis (Fig. 10G)	4
4	Inner face of chela adjacent to articulation with dactylus with two short setulate setae (Fig. 10H). Uropodal endopod with article-2 about 2/3 length of article-1 (Fig. 10B)	*Paratanais euelpis* Barnard, 1920 *sensu* Lang (1973) [Southeast Atlantic: South Africa]
–	Inner face of chela adjacent to articulation with dactylus with single, short setulate seta (Fig. 10I). Uropodal endopod with articles about equal in length (Fig. 10A)	*Paratanais rosadi* sp. n. [Southwest Atlantic: Puerto Rico]

Males are known only for 11 species of *Paratanais* (see Table 1). The male of *Paratanais rosadi* appears to be most similar to that of *Paratanais clarkae* Bird & Bamber, 2000 by having (1) large eyes occupying almost half the length of carapace and (2) antennule with seven articles. The male of *Paratanais rosadi* differs from *Paratanais clarkae* by having (1) a cheliped inner face having a “comb row” of ten small stout setae instead of 12; and (2) a cheliped dactylus without setae on the inner edge. The basis on the male antennule of *Paratanais rosadi* it is very similar to that of *Paratanais puia*, which also has an antennule with seven articles, but differs by (1) the proportions of the carapace; (2) the length of bases of the pereopods; and (3) having a shorter uropodal exopod.

**Table 1.** Alphabetical listing of the 23 currently recognized species for the genus *Paratanais* Dana, 1852, including information on distribution and depth range based on studies by Bird and Bamber (2000, 2013), Araújo-Silva and Larsen (2012), Anderson (2013), and a present study.

Species	Geographical area	Depth range (m)
*Paratanais caterae* Bird & Bamber, 2013*	New Zealand	lntertidal
*Paratanais clarkae* Bird & Bamber, 2000*	Indo-Pacific (South China Sea)	3.5–16
*Paratanais coelhoi* Araújo-Silva & Larsen, 2012	Southwest Atlantic (Brazil)	40
*Paratanais elongatus* (Dana, 1849) [**type species**]**	Indo-Pacific (Philippines),	60
*Paratanais euelpis* Barnard, 1920*	South Atlantic (South Africa)	littoral–231
*Paratanais gaspodei* Bamber, 2005	W. Australia (Esperance)	39-40
*Paratanais hamulus* Bird & Bamber, 2013*	New Zealand	55−141
*Paratanais hessleri* Kudinova-Pasternak, 1985	NE Atlantic (Great Meteor Seamount)	325–470
*Paratanais impressus* Kussakin & Tzareva, 1972*	North Pacific (Kurile Island)	3–50
*Paratanais incomptus* Bird & Bamber, 2013	New Zealand	128−437
*Paratanais maleficus* Larsen, 2001*	Australia (Botany Bay)	4–4.5
*Paratanais martinsi* Bamber & Costa, 2009	NE Atlantic (Azores)	37.8−312
*Paratanais monodi* Makkaveeva, 1971	Red Sea	21–80
*Paratanais oculatus* (Vanhöffen, 1914)	South Atlantic (Magellanic); Indian Ocean (Kerguelen Island); and (?) Brazil	littoral−903
*Paratanais paraoa* Bird, 2011*	New Zealand	shore‒15.5
*Paratanais perturbatius* Larsen, 2001	Australia (Botany Bay)	4–4.5
*Paratanais pseudomartinsi* Larsen, 2012	NE Atlantic (Azores)	312
*Paratanais puia* Bird & Bamber, 2013*	New Zealand	25
*Paratanais rosadi* sp. n.	NW Atlantic (Puerto Rico)	14.9–28
*Paratanais tanyherpes* Błażewicz-Paszkowycz & Bamber, 2012	Australia (Bass Strait)	0−81
*Paratanais tara* Bird, 2011*	New Zealand	shore‒12
*Paratanais vetinari* Bamber, 2005*	Western Australia (Esperance)	20–30
*Paratanais wanga* Bamber, 2008*	Australia (Queensland)	4–29

* Indicates male known.

** See Bamber 1998.

Based on the questionable taxonomic status for five nominal species (i.e. *Paratanais atlanticus* Dollfus, 1897; *Paratanais limicola* Harger, 1878; *Paratanais rigidus* Bate & Westwood, 1868; *Paratanais linearis* Haswell, 1885; and *Paratanais tenuis* Thomson, 1880), we follow Sieg (1983) in considering them as species *incertae sedis*. Due to their inadequate descriptions and dubious taxonomic status, these five species are excluded from Table 1, which presents distribution and depth ranges for the 23 species of *Paratanais* sensu Bird and Bamber (2013) recognized or tentatively recognized herein.

## Supplementary Material

XML Treatment for Paratanais

XML Treatment for Paratanais rosadi
